# Meta-Analysis of Porcine Beige Adipogenesis with Cross-Species CD137-CD137L Molecular Docking: Potential Implications for Pig-to-Human Xenotransplantation

**DOI:** 10.3390/cells15191758

**Published:** 2026-09-27

**Authors:** Zhenhua Guo, Liang Wang, Xinmiao He, Shuo Yang, Hong Ma, Bo Fu, Fang Wang, Di Liu, Dongjie Zhang

**Affiliations:** Institute of Animal Husbandry, Heilongjiang Academy of Agricultural Sciences, Key Laboratory of Combining Farming and Animal Husbandry, Ministry of Agriculture and Rural Affairs, No. 368 Xuefu Road, Harbin 150086, China

**Keywords:** beige adipocyte tissue, CD137, fat, pig, UCP1

## Abstract

**Highlights:**

1. Experimental porcine beige adipogenesis is accompanied by changes in *UCP3*, *CIDEA*, *PGC1α*, *DIO2*, and *CD137*, although the available studies are heterogeneous.

2. Cross-species CD137-CD137L docking is structurally plausible, but predicted binding energies do not demonstrate functional macrophage activation.

3. Direct xenograft and cell-based studies are required before linking adipocyte beiging or CD137 signaling to graft dysfunction or rejection.

**Abstract:**

Breakthroughs in gene-editing technologies have brought pig-to-human xenotransplantation to the clinical forefront. Nevertheless, the micro-scale impacts of the complex pathophysiological milieu within recipients on grafts have long been overlooked by the academic community. Focusing on stromal vascular fraction (SVF) cells and preadipocytes widely present in donor organs, this study explores the potential mechanism underlying beige adipogenesis induced by post-operative anti-inflammatory drugs (e.g., dexamethasone) and surgical stress (norepinephrine), as well as its possible relationship with host immune system activation. The literature search and study-selection reporting were informed by PRISMA 2020. Five information sources were searched, and nine relevant studies on experimentally induced porcine adipose beiging, predominantly in vitro, were included in marker-specific meta-analyses. The meta-analysis showed a higher pooled expression of *CD137*, *CIDEA*, *UCP3*, *PGC1α* and *DIO2* during experimentally induced porcine beige adipogenesis. To explore the structural plausibility of cross-species interaction, molecular docking analysis of the candidate protein CD137 was performed using AlphaFold 3. The predicted binding energies were −9.4 kcal/mol for porcine CD137–human CD137L and −9.0 kcal/mol for the human homologous complex. These computational predictions suggest a structurally plausible interaction, but the results do not demonstrate stronger biological binding, macrophage activation, or immune rejection. Overall, the findings support a hypothesis that porcine adipocyte beiging and CD137-related cross-species interactions may be relevant to xenograft immunometabolism; direct experimental validation in xenotransplantation models is required.

## 1. Introduction

Breakthrough advances in gene-editing technologies have propelled pig-to-human xenotransplantation to the forefront of clinical application, offering substantial promise for alleviating the global crisis of donor organ failure and shortage [1,2,3]. To date, several clinical cases of successful pig-derived liver, kidney and heart transplantation into human recipients have been successively reported [4,5,6,7]. Nevertheless, current clinical attention and academic reports are almost exclusively focused on macroscopic systemic clinical manifestations in recipients, hyperacute graft rejection, and conventional immunosuppressive regimens [8,9]. Regrettably, microscale cellular phenotypic remodeling and metabolic reprogramming of transplanted organs under the complex in vivo pathophysiological milieu of human hosts have long been overlooked by the academic community. Such intracellular alterations within grafts may be insidiously undermining the long-term survival of grafts.

Stromal Vascular Fraction (SVF) cells, core components of vascular endothelium along with preadipocytes residing in donor organs, are highly prevalent and widely distributed in various tissues and organs, including the heart [10,11], kidney [12], and liver [13,14]. Xenotransplantation surgery may trigger beige adipogenesis in these preadipocytes and SVF cells, which has not been documented in previous xenotransplantation studies. The essence of adipocyte beiging is the shift toward a thermogenic pattern with elevated cellular metabolic activity, accompanied by up-regulated expression of certain marker genes. Nevertheless, controversies remain regarding the induction of beiging in porcine adipocytes. Porcine breeds exhibit divergent sensitivities to inducing agents; Duroc–Landrace–Yorkshire (DLY) adipocytes cannot be induced by epinephrine, whereas Min pig adipocytes undergo beiging upon epinephrine stimulation [15]. Adipocytes isolated from different anatomical sites display distinct beiging responses, with differential gene expression after beiging induction in cells derived from back fat and abdominal fat [16]. Following successful adipocyte beiging, subsequent nonivamide treatment reduces *DIO2* expression [17]. Porcine beige-adipocyte studies have used both cell-based induction systems and, less commonly, in vivo models. Their molecular readouts include *UCP3*, *CIDEA*, *PGC1α*, *DIO2*, *CD137* and related markers, while responses can vary with breed, adipose depot, differentiation protocol and inducing stimulus [6,15,16,17]. These differences are important when extrapolating experimental beiging to the xenotransplantation setting.

Given this background, a comprehensive and systematic evaluation of the expression profiles of key marker genes and their underlying protein–protein interaction networks during porcine adipocyte beiging is of critical scientific value for understanding this phenotype. This study aims to evaluate the molecular characteristics of experimentally induced porcine adipocyte beiging and to explore, through cross-species molecular docking, a hypothesis concerning its potential relevance to xenotransplantation-related immune interactions.

## 2. Methods

### 2.1. Database Search Strategy and Study Inclusion

The literature search and study-selection process were reported with reference to PRISMA 2020 as a reporting guideline. PubMed, Web of Science Core Collection, Scopus, ProQuest, and ScienceDirect were searched from database inception to 1 January 2026. PubMed, Web of Science, and Scopus served as the principal bibliographic databases; ProQuest provided an additional multidisciplinary source, and ScienceDirect was retained as an additional full-text discovery source rather than as a substitute for bibliographic databases. Search terms were structured according to the PICOS principle and adapted to the syntax and searchable fields of each source. The core concept combined (beige OR brite), (adipocytes OR fat OR adipogenesis OR “beige adipose tissue”), and (pig OR porcine OR swine OR hog OR boar OR sow OR piglet). Complete database-specific strategies, fields, Boolean syntax, and controlled vocabulary, where applicable, are provided in Supplementary Table S1. The review protocol was not prospectively registered.

### 2.2. Data Extraction

The inclusion and exclusion criteria are presented in Table 1. Two researchers with experience in meta-analysis independently screened records in two stages: primary screening and secondary screening. During primary screening, obviously irrelevant literature was excluded based on titles and abstracts. Full-text articles were assessed in secondary screening to determine compliance with inclusion criteria. Disagreements between the two reviewers were resolved by arbitration from a third author. Reasons for exclusion at each step were documented, and the PRISMA flow diagram (Figure 1) was subsequently generated.

A standardized data-extraction spreadsheet was pre-designed. Two researchers independently extracted data and cross checked the extracted results. Data that were consistent after cross-checking were entered into the database, and discrepant data were reverified against the original articles. Extracted items included first author, publication year, porcine breed, cellular sampling site, cell passage number, induction medium formulation, induction duration, serum concentration, incubation temperature, intervention concentration, sample size, mean value, and standard error. The standard deviation reported in the original papers was uniformly converted to standard error. For gene-expression data presented only in figures, GetData Graph Digitizer software (version 2.26) was adopted for figure data extraction. Corresponding authors were contacted via e-mail for studies with missing data, with a 4-week waiting period. Studies that otherwise met the review criteria but lacked extractable numerical data remained eligible for qualitative synthesis and were excluded only from quantitative pooling when an effect estimate could not be reconstructed.

### 2.3. Methodological Quality Considerations

Because the included evidence comprised predominantly in vitro studies and one in vivo study, no single risk-of-bias tool was directly applicable to all designs. We therefore appraised each study descriptively for experimental replication, definition of the experimental unit, reporting of randomization or blinding where applicable, gene-expression normalization, completeness of outcome reporting, and adequacy of statistical reporting. The study-level assessment is presented in Supplementary Table S2, allowing the methodological concerns identified for each study to be considered when interpreting the pooled evidence. These domains were not converted into a single numerical quality score.

### 2.4. Meta-Analysis

Meta-analysis was performed using Review Manager (version 5.4). Gene-expression outcomes were treated as continuous variables. Because eligible studies reported expression using different normalized scales and experimental platforms, standardized mean differences (SMDs) with 95% confidence intervals (CIs) were used to place results for the same molecular marker on a common standardized scale. Each marker gene was synthesized as a separate outcome; measurements of different genes from the same experiment were not treated as interchangeable outcomes or compared against one another as network nodes. Given the evident biological and methodological diversity in breed, age, tissue source, induction protocol, concentration, and treatment duration, a random-effects model was used because genuine between-study heterogeneity was expected rather than selected according to an *I*^2^ threshold. *I*^2^ was retained as a descriptive measure of heterogeneity.

### 2.5. Protein–Protein Interaction Networks Methods

Differentially expressed genes were extracted after the induction of beige adipocytes from white adipocytes according to the included studies for protein–protein interaction networks (PPI) analysis. Specifically, the obtained genes were imported into STRING (https://cn.string-db.org/, version 12.0, accessed on 1 May 2026) to construct PPI network data. Result visualization was performed using Cytoscape (version 3.7.2).

### 2.6. Molecular Docking of Candidate Proteins

Amino acid sequences of the key candidate protein CD137, screened out via meta-analysis, were downloaded from the National Center for Biotechnology Information website (https://www.ncbi.nlm.nih.gov/, accessed on 7 May 2026), including human CD137 (XM_047419672.1), porcine CD137 (XP_013854397.1), and human CD137L (NM_003811.4). Molecular docking was carried out using AlphaFold 3 (https://alphafoldserver.com, accessed on 7 May 2026). Docking between human CD137 and human CD137L was set as the control group [18,19], and docking of porcine CD137 with human CD137L was simulated to mimic the xenotransplantation microenvironment. The binding affinity (ΔG) between protein complexes derived from docking simulations was calculated via PRODIGY (https://wenmr.science.uu.nl/prodigy, accessed on 7 May 2026). Three-dimensional visual presentation of docking complexes was accomplished with PyMOL software (www.pymol.org, version 3.1, accessed on 7 May 2026).

## 3. Results

### 3.1. Literature Screening and Basic Characteristics of Included Studies

A total of nine eligible studies were finally included in this meta-analysis [6,15,16,17,20,21,22,23,24] (Figure 1A, Table 2). Eight studies were predominantly in vitro and used stromal vascular fraction (SVF) cells, preadipocytes or adipocytes, whereas Xuan et al. [16] evaluated adipose tissue from MSTN-knockout pigs in vivo. Experimental materials originated from dorsal subcutaneous fat and abdominal visceral adipose tissue of pigs aged from 7 days to 7 months. Intervention durations ranged from 1 to 9 days in the cell-based studies. Porcine beige adipocyte marker genes detected across these studies comprised *UCP2, UCP3*, *CIDEA*, *PGC1α*, *PPARγ*, *PRDM16*, *DIO2*, *EBF2*, *CD137* and others.

### 3.2. Meta-Analysis Results

For studies with available direct comparisons, the forest plot results from traditional meta-analysis (Figure 1B) revealed that during the induction of beige adipogenesis in porcine SVF cells, the expression of beige adipocyte marker genes *CD137* (standard MD [SMD], 4.58; 95% CI, 1.89–7.27; *p* < 0.001), *CIDEA* (SMD, 11.6; 95% CI, 4.99–18.21; *p* < 0.001), *PPARγ* (SMD, 9.68; 95% CI, 5.15–14.21; *p* < 0.001), and *UCP3* (SMD, 10.18; 95% CI, 4.54–15.82; *p* < 0.001) were significantly up-regulated, exhibiting a significant positive correlation with porcine adipocyte beiging.

Figure 1C,D results indicated that during the induction of beige adipogenesis in porcine white adipocytes, beige adipocyte marker genes *CD137* (SMD, 1.5; 95% CI, 0.53–2.48; *p* < 0.001), *CIDEA* (SMD, 1.28; 95% CI, 0.38–2.18; *p* < 0.001), *DIO2* (SMD, 2.28; 95% CI, 1.1–3.47; *p* < 0.001), *PGC1α* (SMD, 7; 95% CI, 1.82–12.18; *p* < 0.001), and *UCP3* (SMD, 6.04; 95% CI, 4.13–7.95; *p* < 0.001) were significantly positively correlated with the beiging process. Nevertheless, *PPARγ* (SMD, 0.85; 95% CI, −3.01–4.71; *p* = 0.66) showed no correlation with adipocyte beiging. In addition, heterogeneity tests revealed substantial heterogeneity across included studies (*I*^2^ >50%). Subgroup analysis further identified porcine breed and induction protocols as the main sources of heterogeneity.

### 3.3. Protein–Protein Interaction Networks

A PPI network (Figure 2) was constructed based on genes with significantly altered expression during porcine adipocyte beiging. The six key proteins focused on in this study were located in the central region of the network. In addition, nine proteins, including BMP2 and FAS, failed to be incorporated into this PPI network. Notably, Kim reported the expression changes of porcine UCP1 during adipocyte beiging in one study [6]; therefore, UCP1 was also included in the PPI network.

### 3.4. Comparative Molecular Docking of Human and Porcine CD137

To explore the potential structural basis of a cross-species CD137–CD137L interaction, molecular docking of the candidate protein CD137 was performed in the present study (Figure 3). The docking complex of human CD137–human CD137L (Figure 3A) formed a predicted hydrogen-bond network at the binding interface. Key binding residues included CYS102, LYS69, GLN57, and CYS62 of human CD137, which were predicted to interact with ALA154, VAL152, GLN227, GLN230, GLN98, ALA116, and GLY114 of human CD137L, with a predicted binding affinity of ΔG = −9 kcal/mol. The spatial conformation of the porcine CD137–human CD137L docking complex (Figure 3B) showed similarity to the human homologous complex. Human CD137L involved the same predicted set of binding residues, while porcine CD137 docked via CYS107, GLU74, LYS72, and CYS67, with a predicted ΔG of −9.4 kcal/mol. The 0.4 kcal/mol difference between the predicted energies was not interpreted as evidence of biologically stronger binding, and docking alone cannot demonstrate functional macrophage activation or immune rejection.

## 4. Discussion

### 4.1. The Novel Insight

Successful clinical studies of porcine-to-human xenotransplantation of the liver, kidney and heart have been previously reported [4,5,6,7]. These studies mainly focused on macroscopic systemic clinical manifestations and conventional immune rejection in recipients. Nevertheless, microcellular phenotypic and metabolic remodeling within xenografts in human recipients remains incompletely characterized. In the present study, marker genes involved in porcine beige adipogenesis were evaluated using marker-specific meta-analyses and protein–protein interaction. We propose that adipogenic cells within donor organs may have the potential to undergo beige-like remodeling under complex pathophysiological and pharmacological conditions after xenotransplantation. This possibility has not been directly demonstrated in xenografts and should therefore be regarded as a testable hypothesis rather than an established mechanism of graft dysfunction.

### 4.2. Mechanisms of Drug-Mediated Adipocyte Beiging

Recipients usually receive high-dose glucocorticoids such as dexamethasone to suppress acute inflammation and rejection during peri-operative and post-transplant monitoring periods of xenotransplantation [25,26]. Meanwhile, circulating norepinephrine levels can increase owing to surgical stress [27,28,29]. In the included in vitro studies, dexamethasone was generally used as one component of a complex adipogenic differentiation cocktail rather than as an isolated beiging stimulus [6,20,23], and subsequent norepinephrine stimulation was used in some protocols to promote a beige phenotype [15,21] (Table 2). Therefore, these experimental protocols do not demonstrate that clinically relevant dexamethasone or postoperative norepinephrine exposure would induce the same phenotype in xenografts; direct studies using physiologically relevant concentrations and exposure conditions are required.

### 4.3. Cascade Injury of Grafts Triggered by Adipocyte Beiging

The gold-standard hallmarks of beiging in porcine adipocytes consist of substantial mitochondrial proliferation and abundant multilocular lipid droplet formation [30]. From a purely metabolic perspective, this cellular transformation is accompanied by a step-wise up-regulation of uncoupling proteins such as *UCP3*, reprogramming cells from an energy storage phenotype toward a thermogenic and high-energy-consuming metabolic state [6]. Meanwhile, several key marker genes including *CD137*, *CIDEA*, *PGC1α*, *DIO2* and *PPARγ* are significantly elevated [22,24]. Our marker-specific meta-analyses likewise showed higher pooled expression of several beiging-associated markers during experimentally induced porcine adipogenesis (Figure 1).

Macrophages exert important regulatory effects during adipocyte beiging [31,32,33,34]. The transformation of white adipose tissue into beige adipose tissue has been associated with tissue remodeling, and M2 macrophage polarization can facilitate thermogenic responses in some experimental settings [31]. Macrophage abundance may also increase during this process [35]. These observations raise the possibility that immune stress and metabolic remodeling could interact in xenotransplantation; however, the present meta-analysis did not directly assess pyroptosis, systemic inflammatory response syndrome, or macrophage-mediated graft injury. Any such pathway therefore remains hypothetical and requires direct experimental validation. A further exploratory component of the present study was the AlphaFold 3 molecular docking combined with PRODIGY binding-affinity prediction. The predicted binding energy for porcine CD137–human CD137L was ΔG = −9.4 kcal/mol, compared with ΔG = −9.0 kcal/mol for the human homologous complex (Figure 3). The small difference between these computational estimates should not be interpreted as evidence that the cross-species interaction is biologically stronger. Rather, the docking result suggests structural plausibility for a porcine CD137–human CD137L interaction. Whether porcine CD137 expressed during adipocyte beiging can bind CD137L in a cellular context, modify human macrophage activation, or influence xenograft function remains to be tested experimentally.

### 4.4. Limitations

This study has several limitations. First, only nine eligible studies were available, eight of which were predominantly in vitro, and the studies differed substantially in breed, age, adipose/cellular source, induction protocols, concentrations, and treatment durations. Second, adipocyte beiging has not been directly demonstrated in pig-to-human xenografts, and the current evidence cannot establish a causal relationship between porcine adipocyte beiging and graft dysfunction or rejection. Third, molecular docking is predictive rather than confirmatory and does not establish functional activation of human macrophages by porcine *CD137*. Fourth, methodological reporting was incomplete for several domains, including randomization, blinding, and experimental-unit definition; the study-specific appraisal is provided in Supplementary Table S2. Fifth, the review protocol was not prospectively registered, and eligibility and analytical decisions clarified during peer review should therefore be interpreted transparently. These limitations reduce the certainty with which the pooled findings can be extrapolated beyond the experimental systems studied.

### 4.5. Future Perspectives

Future studies should directly test the proposed hypothesis in xenotransplantation-relevant models. Priority experiments include determining whether adipocyte beiging occurs within xenografts, testing clinically relevant glucocorticoid and catecholaminergic exposures, validating porcine CD137–human CD137L interaction using cellular and biochemical assays, and assessing whether this interaction alters human macrophage activation. If these steps are experimentally supported, subsequent studies could evaluate whether modulation of *UCP3*, *CD137* or adipocyte beiging changes graft metabolism, function or survival.

## 5. Conclusions

This meta-analysis indicates that experimentally induced porcine beige adipogenesis is accompanied by changes in several molecular markers, including *CD137*, *CIDEA*, *UCP3*, *PGC1α* and *DIO2*. Marker-specific pooled estimates varied across genes and experimental systems and were interpreted separately rather than ranked against one another. Cross-species docking suggested structural plausibility for the porcine CD137–human CD137L interaction, but it does not demonstrate functional macrophage activation. The possible relevance of adipocyte beiging to pig-to-human xenotransplantation should therefore be considered a hypothesis that requires direct experimental validation.

## Figures and Tables

**Figure 1:**
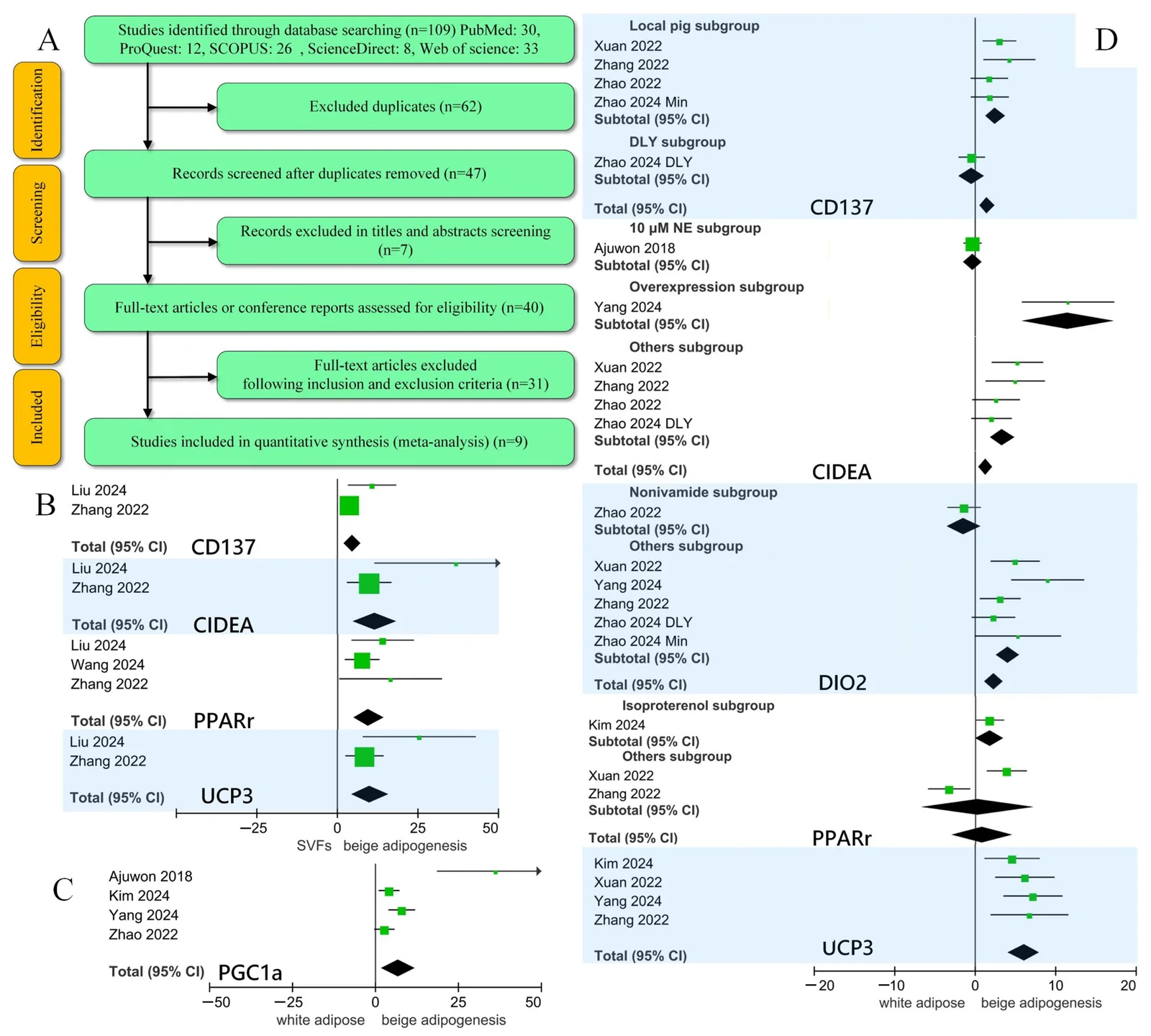
Summary of the study selection process and traditional meta-analysis. (**A**). Study selection process. A total of 109 pieces of literature were retrieved from five databases according to predefined keywords, and 9 studies were finally included after screening. (**B**). Forest plot of marker-gene expression during beige adipogenesis induced in porcine SVF cells. (**C**,**D**). Forest plots of marker-gene expression during beige adipogenesis induced in porcine white adipocytes.

**Figure 2:**
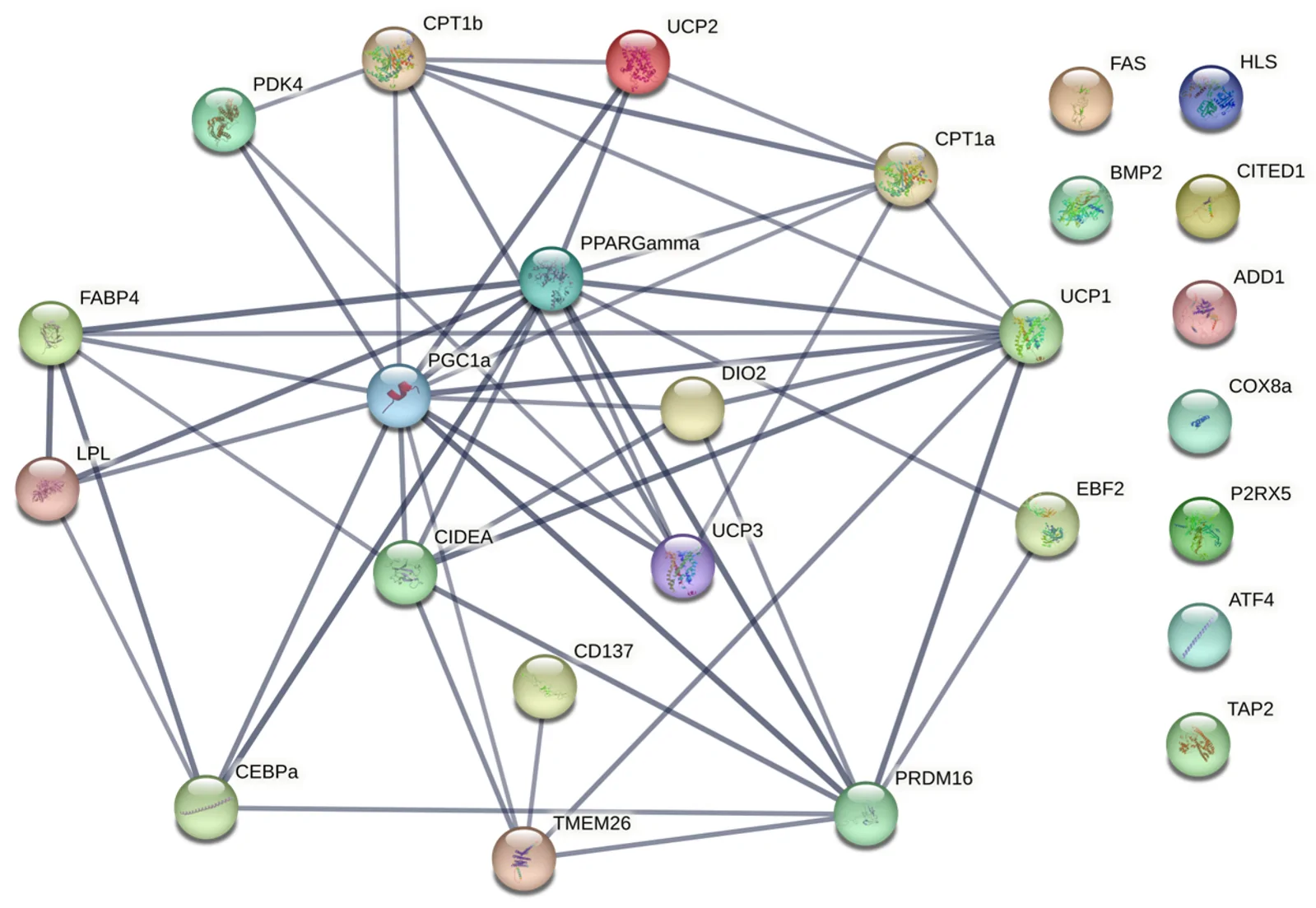
Protein–protein interaction networks of induced beige adipocytes. Protein conformations shown in the figure were generated from respective PDB files using PyMOL software (version 3.1, www.pymol.org).

**Figure 3:**
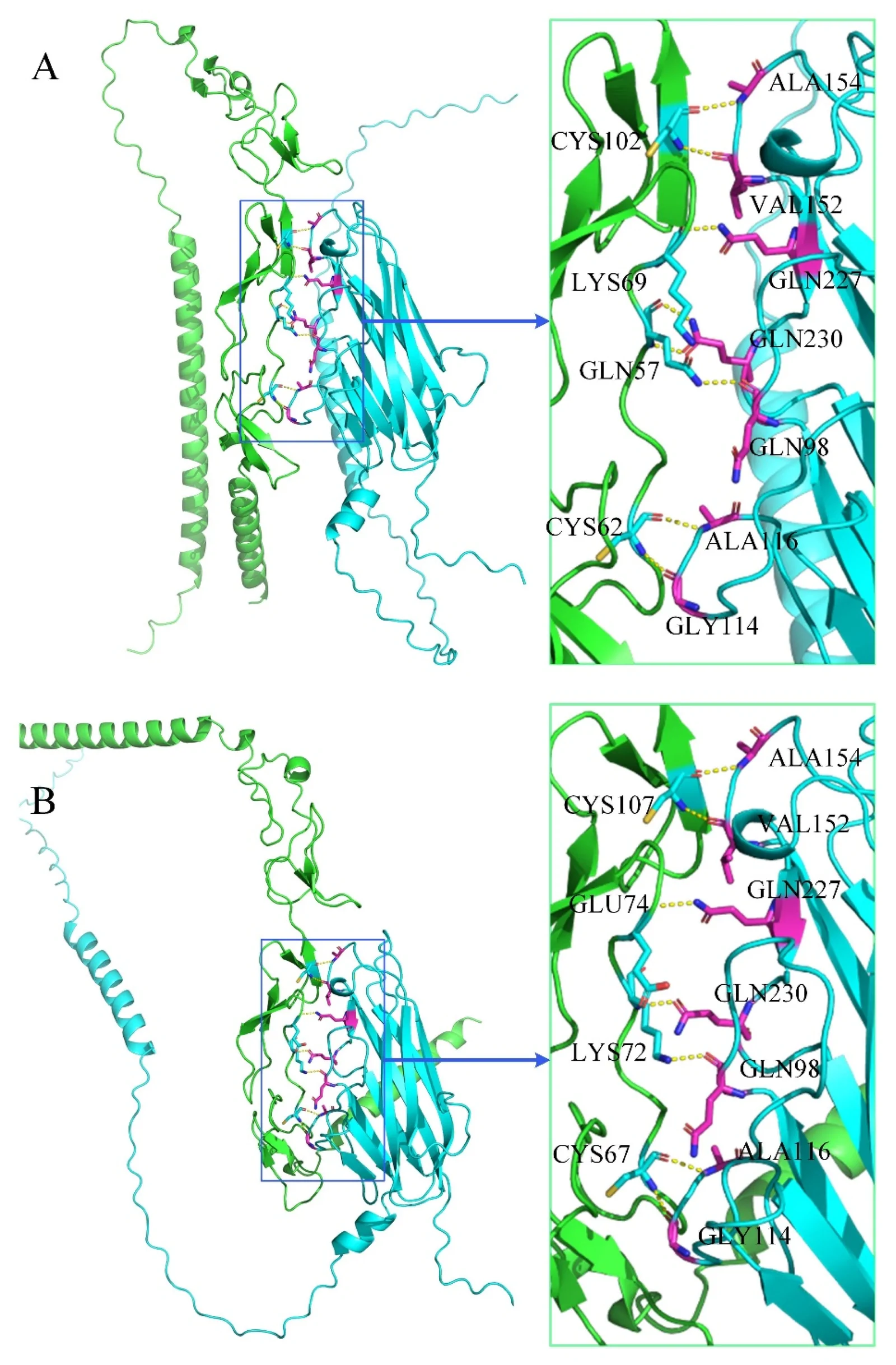
Comparative molecular docking of CD137. (**A**). Docking of human CD137 with human CD137L. (**B**). Docking of porcine CD137 with human CD137L. CD137 proteins are shown in green chains, and CD137L is shown in blue chains. Amino acid residues of CD137 within the binding pocket are displayed as blue sticks for residues with distances less than 3 Å (Ångstrom), while residues of CD137L are presented as purple sticks.

**Table 1:** Inclusion and exclusion criteria.

Inclusion	Exclusion
Species evaluated included but were not limited to pigs	Pigs were not used
English literature	Non-English
Beiging-specific gene expression (*UCP3*, *CIDEA*) alone or in combination with other genes in pigs	No eligible beiging-related molecular outcome assessed
Adipocyte tissue data included	No adipocyte tissue data

**Table 2:** Characteristics of studies included.

	Study Year	Breed	Age	Tissue	Adipocyte Differentiation Inducement	Beige Adipogenesis Inducement	Treat Time	Genes	Control vs. Treatment
1	Shin 2018 [21]	DLY pig	Not mentioned	Preadipocytes in vitro	1.7 μM INS, 1 μM Dex, 1 μM RSG, 17 μM pantothenic acid, 33 μM biotin, 1 nM T3	10 μM NE	9 days	*CIDEA*, *PGC1α*, *UCP1*, *PRDM16*, *CPT1a*	WA vs. BA
2	Kim 2024 [6]	Not mentioned	5–7 months old	Adipocytes in vitro	10 μg/mL INS, 1 nM T3, 10 μg/mL transferrin, 1 μM RSG, μM Dex	1 μM isoproterenol	6 days	*UCP2*, *UCP3*, *PPARγ*, *PGC-1a*, *UCP1*	WA vs. BA
3	Liu 2024 [20]	Tibetan pig	1-month-old male	SVFs in vitro	1 nM T3, 1 μM Dex, 0.5 mM IBMX 2 μM RSG	Same as adipocyte differentiation	8 days	*UCP3*, *PPARγ*, *PGC1α*, *CIDEA*, *BMP2*, *EBF2*, *CITED1*, *P2RX5*, *CD137*, *FABP4*, *CEBPa*	SVFs vs. BA
4	Wang 2024 [23]	Min pig	7-day-old male	SVFs in vitro	INS 10 μg/mL, Dex 1 μM, IDM 0.125 mM, IBMX 0.5 mM, T3 1 nM, RSG 1 μM.	Not mentioned	6 days	*PPARγ*, *TAP2*, *EBPb*, *ADD1*, *HLS*, *EBPa*, *FAS*, *LPL*	SVFs vs. BA
5	Xuan 2022 [16]	*MSTN* knockout pig	6 months old	Fat tissues in vivo	Not mentioned	*MSTN* knockout pigs	6 months old	*UCP3*, *PPARγ*, *CIDEA*, *TMEM26*, *CD137*, *DiO2*, *CycS*, *Cox3*, *MCAD*	WA vs. BA
6	Yang 2024 [24]	Min pig	1 month old	SVFs in vitro	1 nM T3, 0.5 μM INS, 2 μM RSG	Overexpression ADRB3	1 day	*PPARγ*, *CEBPa*, *FABP4*, *PGC1-a*, *UCP3*, *Dio2*, *CIDEA*, *COX7A1*, *EBF2*, *PDK4*, *TMEM26*, *LEP*, *ADCY5*, *ADIPOQ*, *HOXC8*, *HOXC9*	WA vs. BA
7	Zhang 2022 [22]	Tibetan pig	1-month-old male	SVFs in vitro	5 μg/mL INS, 17 μM pantothenate, 33 μM biotin, 1 μM Dex, 0.25 mM IBMX 50 mM RSG	1 nM T3, 1 μM Dex, 0.5 mM IBMX 2 μM RSG	WA for 5 days, BA for 8 days	*UCP3*, *PDK4*, *CIDEA*, *EBF2*, *DIO2*, *CD137*, *PPARγ*	SVFs vs. BA, WA vs. BA
8	Zhao 2022 [17]	Erhualian pigs	8-day-old male	Preadipocytes in vitro	0.25 nM Dex, 0.1 mM IBMX, 5 mg/mL INS	25 μM nonivamide	8 days	*CD137*, *TMEM26*, *SCL27A1*, *PRDM16*, *CIDEA*, *DIO2*, *ATF4*, *PGC1α*, *CPT1b*, *COX8a*	WA vs. BA
9	Zhao 2024 [15]	Min pig, DLY pig	7-day-old male	SVFs in vitro	5 μM Dex, 5 μg/L INS, 0.1 mM IBMX, 125 μM IDM, 1 μM RSG, 1 nM T3	NE (concentration not mentioned)	4 days	*DIO2*, *CD137*, *CIDEA*, *TMEM26*, *TOM20*, *PPARγ*	WA vs. BA

Liothyronine (triiodothyronine) is T3; rosiglitazone is RSG; indomethacin is IDM; dexamethasone is Dex; 3-isobutyl-1-methylxanthine is IBMX; insulin is INS; Duroc–Landrace–Yorkshire is DLY; white adipocyte is WA; beige adipocyte is BA; norepinephrine is NE; stromal vascular fraction cells are SVFs; and the *myostatin* gene is *MSTN*. Genes list PCR results only.

## Data Availability

Please contact the author to request the data.

## Supplementary Materials

The following supporting information can be downloaded at https://www.mdpi.com/article/10.3390/cells15191758/s1: Supplementary Table S1 provides database-specific search strategies. Supplementary Table S2 presents the study-level methodological quality appraisal used to contextualize the pooled evidence.
