# Supplementary material for: Meta-Analysis of Porcine Beige Adipogenesis with Cross-Species CD137-CD137L Molecular Docking: Potential Implications for Pig-to-Human Xenotransplantation
Source: Cells. 2026 Sep 27;15(19):1758. doi: 10.3390/cells15191758 (PMC13664533; doi:10.3390/cells15191758)
Supplement: Supplementary file 1 [file cells-15-01758-s001.zip › cells-4528260-supplementary.pdf]

cells

MDPI

1 Supplementary Materials

Supplementary Table S1 provides database-specific search strategies.

Supplementary Table S2 presents the study-level methodological quality appraisal used to contextualize the pooled evidence.

Supplementary Table S1. Database-specific literature-search strategies.

Database / fields	Exact search syntax	Role in search
PubMed; Title/Abstract + MeSH where applicable	((beige[Title/Abstract] OR brite[Title/Abstract]) AND (adipocyte*[Title/Abstract] OR fat[Title/Abstract] OR adipogenesis[Title/Abstract] OR "beige adipose tissue"[Title/Abstract] OR "Adipocytes"[Mesh]) AND (pig[Title/Abstract] OR porcine[Title/Abstract] OR swine[Title/Abstract] OR hog[Title/Abstract] OR boar[Title/Abstract] OR sow[Title/Abstract] OR piglet[Title/Abstract] OR "Swine"[Mesh]))	Principal bibliographic database
Web of Science Core Collection; Topic (TS)	TS=((beige OR brite) AND (adipocyte* OR fat OR adipogenesis OR "beige adipose tissue") AND (pig OR porcine OR swine OR hog OR boar OR sow OR piglet))	Principal bibliographic database
Scopus; TITLE-ABS-KEY	TITLE-ABS-KEY((beige OR brite) AND (adipocyte* OR fat OR adipogenesis OR "beige adipose tissue") AND (pig OR porcine OR swine OR hog OR boar OR sow OR piglet))	Principal bibliographic database
ProQuest; Title/Abstract/Subject	(beige OR brite) AND (adipocyte* OR fat OR adipogenesis OR "beige adipose tissue") AND (pig OR porcine OR swine OR hog OR boar OR sow OR piglet)	Additional multidisciplinary source
ScienceDirect; title, abstract or author-specified keywords	(beige OR brite) AND (adipocytes OR fat OR adipogenesis OR "beige adipose tissue") AND (pig OR porcine OR swine OR hog OR boar OR sow OR piglet)	Additional full-text discovery source; not a substitute for bibliographic databases

*Searches covered database inception to 1 January 2026; syntax was adapted to the searchable fields available in each information source.

Supplementary Table S2. Study-level methodological quality appraisal.

Study	Design	Replication / experimental unit	Randomization / blinding	Normalization / reporting	Main methodological concern
Shin 2018	In vitro	Replication reported; unit partly clear	N/A or NR / NR	Reported / Reported	Biological versus technical replication not always explicit.
Kim 2024	In vitro	Replication reported; unit partly clear	N/A or NR / NR	Reported / Reported	Cell-culture replication and independence require cautious interpretation.
Liu 2024	In vitro	Replication reported; unit partly clear	N/A or NR / NR	Reported / Reported	Biological versus technical replication not always explicit.

Wang 2024	In vitro	Replication reported; unit partly clear	N/A or NR / NR	Reported / Reported	Experimental-unit definition incompletely detailed.
Xuan 2022	In vivo	Animal-level unit reported	NR / NR	Reported / Reported	Randomization and blinding not clearly reported.
Yang 2024	In vitro	Replication reported; unit partly clear	N/A or NR / NR	Reported / Reported	Cell-culture replication and independence require cautious interpretation.
Zhang 2022	In vitro	Replication reported; unit partly clear	N/A or NR / NR	Reported / Reported	Multiple molecular outcomes from the same experimental system.
Zhao 2022	In vitro	Replication reported; unit partly clear	N/A or NR / NR	Reported / Reported	Cell-culture replication and independence require cautious interpretation.
Zhao 2024	In vitro	Replication reported; unit partly clear	N/A or NR / NR	Reported / Reported	Breed-specific experiments and cell-culture replication limit direct comparability.

*NR, not reported; N/A, not applicable. The appraisal was descriptive and was not converted into a numerical quality score.

https://doi.org/10.3390/xxxxx
